# Dare to be resilient: the key to future pesticide-free orchards?

**DOI:** 10.1093/jxb/erae150

**Published:** 2024-04-18

**Authors:** Marie Serrie, Fabienne Ribeyre, Laurent Brun, Jean-Marc Audergon, Bénédicte Quilot, Morgane Roth

**Affiliations:** INRAE, UR GAFL, Avignon, France; CIRAD, UMR PHIM, Montpellier, France; INRAE, UERI Gotheron, Saint-Marcel-Lès-Valence, France; INRAE, UR GAFL, Avignon, France; INRAE, UR GAFL, Avignon, France; INRAE, UR GAFL, Avignon, France; University of Ghent, Belgium

**Keywords:** Biomarkers, breeding, disease resilience, fruit tree, pesticide reduction, pests and diseases, sustainability

## Abstract

Considering the urgent need for more sustainable fruit tree production, it is high time to find durable alternatives to the systematic use of phytosanitary products in orchards. To this end, resilience can deliver a number of benefits. Relying on a combination of tolerance, resistance, and recovery traits, disease resilience appears as a cornerstone to cope with the multiple pest and disease challenges over an orchard’s lifetime. Here, we describe resilience as the capacity of a tree to be minimally affected by external disturbances or to rapidly bounce back to normal functioning after being exposed to these disturbances. Based on a literature survey largely inspired from research on livestock, we highlight different approaches for dissecting phenotypic and genotypic components of resilience. In particular, multisite experimental designs and longitudinal measures of so-called ‘resilience biomarkers’ are required. We identified a list of promising biomarkers relying on ecophysiological and digital measurements. Recent advances in high-throughput phenotyping and genomics tools will likely facilitate fine scale temporal monitoring of tree health, allowing identification of resilient genotypes with the calculation of specific resilience indicators. Although resilience could be considered as a ‘black box’ trait, we demonstrate how it could become a realistic breeding goal.

## Teaser

Based on a multidisciplinary literature review, we contextualize, reflect on, and propose a novel breeding perspective on disease resilience to deal with the massive pesticide restrictions in fruit production.

## Introduction

Plant pests and diseases are responsible for massive yield and economic losses and are a global threat for food safety and security (Savary *et al.*, 2019). The diversity of endemic organisms that threaten agricultural crops includes a large range of viruses, bacteria, fungi, phytoplasma, nematodes, and pests. Since these biotic stresses can occur simultaneously throughout the season, this means that plants must face a multi-disease challenge, where possible co-infections have the potential to considerably affect growth and productivity (Savary *et al.*, 2017). On the other hand, due to the large spread of high-yield and genetically homogeneous cultivars for most crops, some major genetic resistances have already been overcome (Gessler *et al.*, 2006; Gibson and Nguyen, 2021). This has led to devastating epidemics in the last decades both for annual and perennial crops, as shown by viral epidemics of cereal yellow dwarf or citrus tristeza (Jones, 2021). Moreover, the current global warming strongly favours the emergence and the re-emergence of pests and pathogens, and can amplify their impact on crops by modifying their biological reactions (Ladányi and Horváth, 2010; Das *et al.*, 2011; Skendžić *et al.*, 2021). On aphids for example, more species are being observed in orchards as well as a higher number of reproduction cycles per year (Hullé *et al.*, 2010; Devi *et al.*, 2019). This unpredictability means that although we know that biotic pressures on crops are likely to intensify in the next few years, they might be difficult to forecast accurately.

Since fruit trees are perennials, pathogen attacks in a single year are likely to affect tree health for the following years. In addition, long rotation time favours the settlement of plant pests and diseases within orchards. The cumulative effect of the different pests and diseases over the years is therefore much more pronounced in fruit trees when compared with annual crops. For producers, this implies that it is not enough to sustain tree health in a single year, but the entire lifetime of the orchard has to be accounted for. Nowadays this multiyear challenge is largely addressed by phytosanitary treatments, and efficient alternatives remain very limited (Lamine *et al.*, 2017). For comparison, the treatment frequency index (TFI) was 29.5 and 18.4, respectively, for apple and peach in 2018 in France (Desprat *et al.*, 2021), which is two to six times higher than values reported in grain (TFI=4.6) and vegetable crops (TFI=7.5, Crisan, 2019; Chapelle, 2023). It should also be highlighted that a significant proportion of phytosanitary products used in orchards are dictated by fresh-market demands, which require flawless fruit with long-conservation ability. All these facts explain why the fruit tree sector is highly dependent on phytosanitary products, and this raises several concerns.

Firstly, not every disease or pest can be cured by pesticides, such as sharka disease caused by *Plum pox* virus (García and Cambra, 2007) or European stone fruit yellows caused by the bacterium *Candidatus* Phytoplasma prunorum (Sauvion *et al.*, 2012). Besides, the massive use of phytosanitary products accelerates the emergence of resistant pests and pathogens (Kole *et al.*, 2019). In any case, the negative impact of pesticides on environmental and human health is largely acknowledged (Wilson and Tisdell, 2001) and society is urging a drastic reduction of pesticides (Du *et al.*, 2017). To address this concern, many active compounds have already been banned (Donley, 2019), and the trend is intensifying in Europe with the ‘Farm to Fork’ strategy (European Commission, 2020). In addition, stricter application procedures are being imposed on farmers: for instance, in France restrictions aimed at protecting bees and other pollinators over flowering periods (Ferreira *et al.*, 2021). Finally, if many biocontrol products (such as essential oils, antimicrobial products, or chemical mediators like pheromones and kairomones) have been developed for orchards, they are still often less efficient and costlier than synthetic products (Nicot *et al.*, 2012). All these points clearly highlight the long-term dead end of phytosanitary products in durably fighting pests and diseases as well as the urgent need to find alternative and sustainable solutions to these products in orchards.

**Fig. 1.**
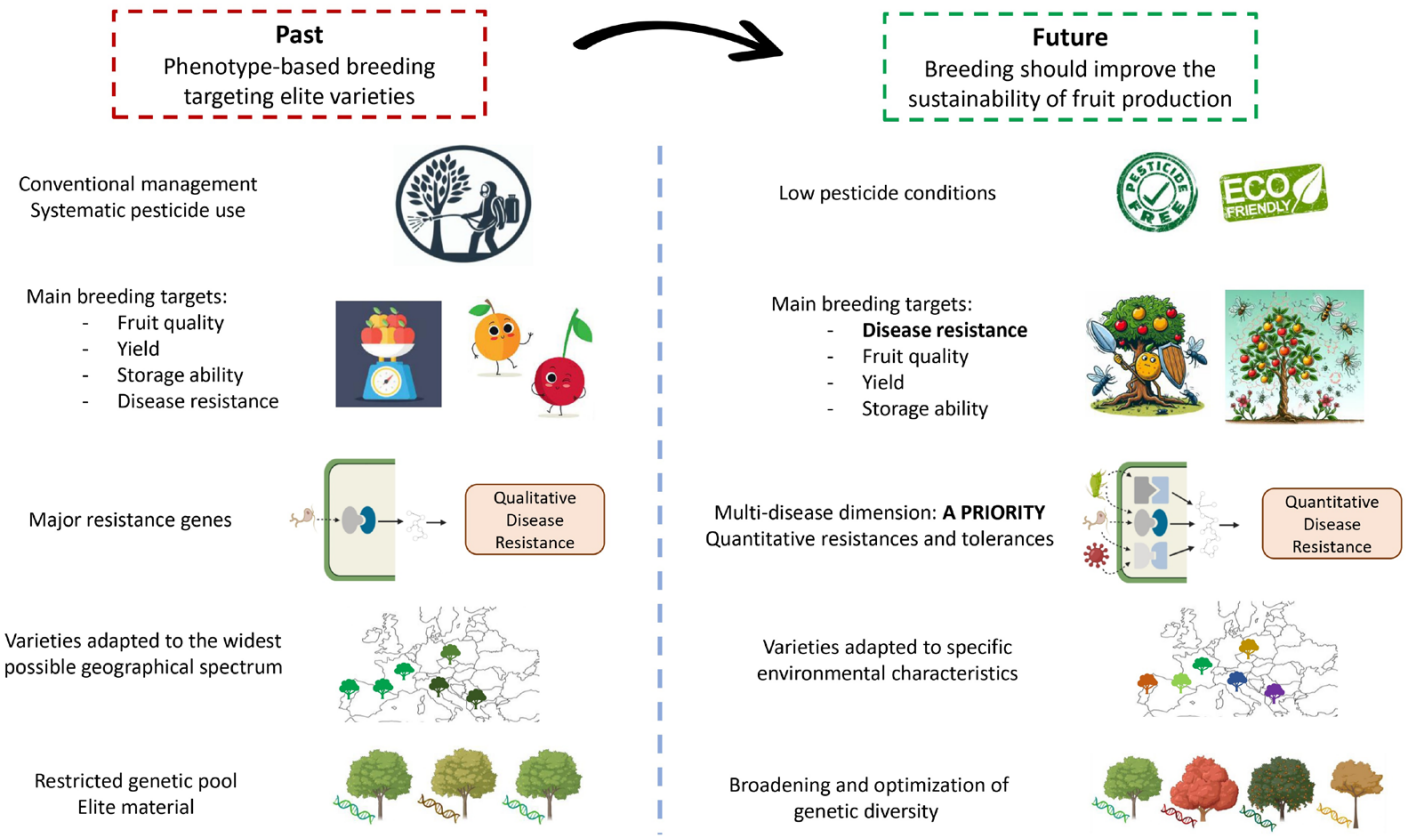
Description of the major shifts recommended for a transition towards a more sustainable breeding strategy accounting for the current environmental and societal constraints.

Among available alternatives, breeding trees for pest and disease resistance is an efficient strategy to cope with biotic pressures. For instance some resistance mechanisms have already been identified for sharka and bacterial canker in apricot (Lambert *et al.*, 2009; Rubio *et al.*, 2014; Omrani *et al.*, 2019), and for aphids and powdery mildew in peach (Sauge *et al.*, 2012; Lambert *et al.*, 2016; Pascal *et al.*, 2017; Duval *et al.*, 2022). Likewise, in apple, the pyramiding of functionally different major resistance genes has shown great efficiency in fighting individually scab, powdery mildew, or fire blight (Baumgartner *et al.*, 2015). We should also acknowledge the emergence in the last decade of a few breeding programmes targeting multiple disease resistance (Wiesner-Hanks and Nelson, 2016). In grapevine, new varieties combining polygenic resistance to both grapevine downy mildew and powdery mildew have been successfully created within the programme ‘ResDur’ (Schneider *et al.*, 2019). However, so far in fruit trees, breeding for low susceptibility to diverse pests and diseases has often been overlooked. Breeding programmes are mostly based on a restricted elite material, targeting varieties suitable to conventional management in the widest possible range of locations. As a result, we still lack elite materials that combine multiple resistances and that would be adapted to low phytosanitary protection. To truly tackle the agroecological transition, a paradigm shift is necessary in fruit tree breeding to prioritize natural defences of trees against pests and diseases (Fig. 1). Unfortunately, the sources of genetic resistance are rare, often come from wild or closely related species, and are difficult to precisely detect and introgress, especially if they have a polygenic basis. Moreover, it has been recognized that the explicit breeding for multiple resistance requires systematic and exhaustive recording of pathogen burden at individual level throughout the time of infection (Mulder and Rashidi, 2017). Unfortunately, these data are not currently recorded in orchards, either because it is particularly laborious and costly, or because adequate inoculation protocols are lacking. If we push this reflection further, considering that new pests or diseases and alternative strains will continue to emerge or re-emerge through the years in a way that is difficult to predict in the context of climate change, and considering the possible bypass of plant resistances, targeting multiple resistance may actually not be sufficient. In addition, it has been shown that a drastic reduction of the phytosanitary umbrella could lead to the reappearance of forgotten diseases, which raises serious concerns for the future. Considering all these constraints, and the fact that in traditional breeding it takes 20 years on average to register a new fruit variety, during the time needed to combine multiple diseases resistances one after the other, the obtained varieties might be already outdated. We thus claim that targeting multi-resistance might not be compatible with the need to rapidly find alternative solutions to pesticides and that, consequently, new breeding targets must be envisioned that go beyond the idea of multiple resistances.

## Searching for the right breeding target: disease resilience as a promising approach

### Disease resilience: resistance, tolerance, and recovery interaction

As illustrated by the multiple, sometimes antagonistic definitions that have emerged over the years, resilience is an attractive concept but it remains difficult to define. This ambiguity originates from a widespread use of the term ‘resilience’ to describe phenomena in multiple disciplines across biology, physics, and social sciences (Martin-Breen and Anderies, 2011). Besides, the fact that resilience is used at multiple scales and can describe properties of an isolated object, an individual, or even of complex multi-organism or multi-actor socio-economic systems adds to the complexity and plasticity of this term. As recommended by Martin-Breen and Anderies (2011), we need to clarify the nature of what is being considered in our context. In our geneticists’ view, we focus on the scale of individual trees or genotypes. A broad definition of resilience, also called general environment resilience, has emerged in the last years and could be relevant here. General environment resilience is defined as the capacity of an organism to be minimally affected by a disturbance or to rapidly return to the physiological, behavioural, cognitive, health, affective, and production states that pertained before exposure to a disturbance (Colditz and Hine, 2016). Interestingly, this concept is also often referred to as ‘robustness’, which is more commonly used to describe the combination of a high production potential with high resilience (Knap and Doeschl‑Wilson, 2020). More specifically, general environment resilience represents the capacity of an organism to maintain its productivity in a wide range of environments, including stressful conditions, without compromising reproduction, health, and wellbeing (Knap, 2005; Urruty *et al.*, 2016). It is thus a composite trait encompassing a variety of profiles in response to different kinds of perturbations (e.g. disease, heat stress, drought).

Bearing in mind the above-described context, and for the sake of clarity, in the present work the focus will be on ‘disease resilience’, as derived from the definition of general environment resilience (Albers *et al.*, 1987; Bisset and Morris, 1996; Bishop, 2012; Doeschl-Wilson *et al.*, 2021). It is often admitted that disease resilience captures two complementary host defence mechanisms, resistance and tolerance, which are defined as follows:

- Disease resistance is the ability of the individual to inhibit or limit within-host pathogen load either by preventing infection in the first place or by inhibiting within-host pathogen replication once infected (Agrios, 2005; Råberg *et al.*, 2008; Bishop, 2012; Knap and Doeschl‑Wilson, 2020).
- Disease tolerance is the ability of an infected host to reduce the impact of this infection on performance and health, i.e. maintaining high health or production performance at a given within-host pathogen load without necessarily reducing this pathogen load (Agrios, 2005; Knap and Doeschl‑Wilson, 2020). A tolerant organism maintains its performance despite the pathogen burden (Mulder and Rashidi, 2017).

In summary, disease resistance is the ability to reduce and control pathogen load whereas disease tolerance is the ability of an infected host to limit the damage caused by a given within-host pathogen load without necessarily reducing this pathogen load.

**Fig. 2.**
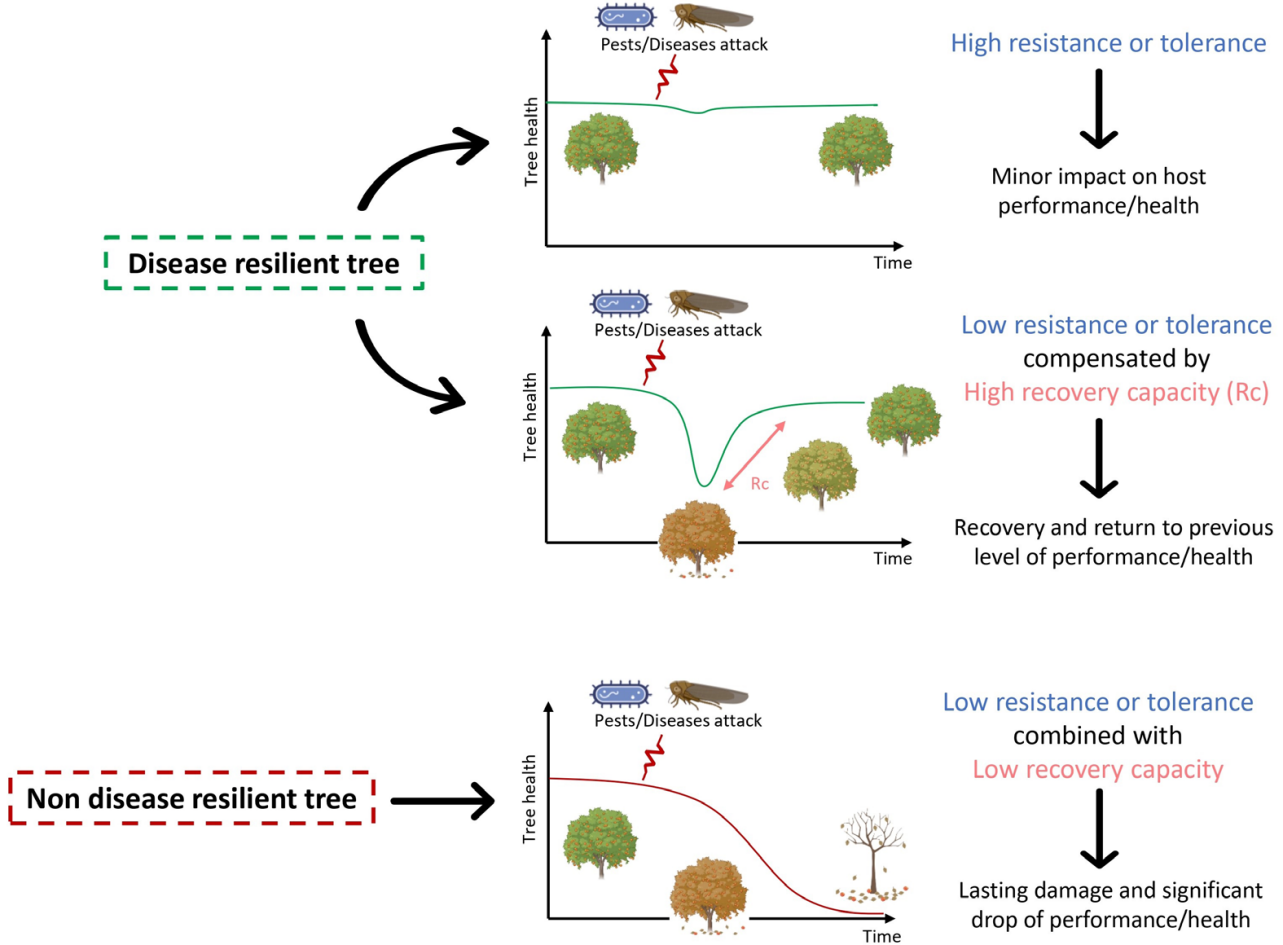
Conceptual illustration of disease resilience expression over time in fruit trees by distinction of (A) the multiple trajectories leading to disease resilience and (B) the decay of non-disease-resilient trees in a multi-disease context.

In the context of fruit tree breeding, we define disease resilience as the capacity of a given individual (i.e. a genotype) to be minimally affected by one or multiple attacks of pests or diseases, and thus to reduce their impact via resistance or tolerance mechanisms, but also to bounce back to normal functioning after being exposed to these disturbances. This ability to return to pre-disturbance levels is achieved through recovery mechanisms reflected in a dynamic manner by high elasticity and a low return time (Fig. 2, see also Lloret *et al.*, 2011; Hodgson *et al.*, 2015; DeSoto *et al.*, 2020). Therefore, we recommend describing disease resilience by quantifying together *resistance*, *tolerance*, and *recovery*. This definition accounts for the fact that disease resilience can follow multiple trajectories that could therefore be governed by different genetic mechanisms. We think that this comprehensive view is necessary for setting up durable fruit tree production under low phytosanitary protection.

### Beyond multi-resistance: the added value and the opportunity of disease resilience

In practice, disease resilience can be used as a ‘black box trait’ that can be targeted without an individual monitoring of pathogen load and without dissecting components of resistance, tolerance, and recovery (Mulder and Rashidi, 2017; Knap and Doeschl‑Wilson, 2020). Until now, most studies on fruit trees have focused on the search for resistance genes for a specific pathogen (Fig. 1). The multi-disease context has not been considered as such, thus ignoring interactions between pests and diseases as well as cumulative effects of multiple attacks and overlooking possible recovery capacity of trees (Khan and Korban, 2022). Paying more attention to trees’ ability to recover would bring a more integrative and long-term vision of plant health. Thus, breeding for disease resilience appears to be a pragmatic and complementary way for fruit tree breeders to cope with the complex, fluctuating, and unpredictable multi disease and pest challenge. The question remaining is how to measure disease resilience in practice. In perennial species, much work has been carried out over the last decades on so-called forest resilience (Thompson *et al.*, 2009; Nikinmaa *et al.*, 2020). If these studies were to be particularly relevant for searching metrics of resilience (Lloret *et al.*, 2011; DeSoto *et al.*, 2020), due to several considerations, they cannot be directly translated into our study system. Chiefly, resilience is there considered at the level of forests, regarded as systems where interspecific interactions are at play, whereas in our case the focus is made up at the genotypic level within mono-specific orchards. Besides, another important difference between forestry and fruit production is that cultural practices, such as pruning and fertilization, strongly determine the expression of traits in fruit trees. Caution is therefore required before transferring knowledge on forest resilience to the case of disease resilience for fruit tree breeding.

### Breeding for disease resilience: a source of inspiration in livestock

Although the methodology for disease resilience breeding does not yet exist for fruit trees, the animal field provides promising sources of inspiration when considering the recent flourishing literature related to disease resilience in livestock breeding. While searching for solutions to fruit tree-oriented matters in the field of livestock breeding may seem surprising at first sight, it turns out that many useful parallels can be drawn between these two worlds. Firstly, in fruit tree breeding as in animal breeding, the focus is on the individual scale where each tree/animal is considered as a unit with a respective genotype. Secondly, fruit trees and livestock must both face the multi-disease challenge and their health must be managed on a multi-year basis, over and after a long juvenile phase. Thirdly, until today vaccines and antimicrobials were massively used to control livestock infection levels, which poses similar problems to phytosanitary products in fruit production in terms of long-term efficiency (microbial/pest resistances, new pathogen emergence) and societal acceptance (antibiotics/vaccines/pesticide residues in our food and in the environment). In both sectors, reducing the dependency on synthetic products is thus perceived as an urgent need.

Over the last few years, more and more studies have specifically addressed the question of breeding for disease resilience in livestock (Colditz and Hine, 2016; Mulder and Rashidi, 2017; Berghof *et al.*, 2019b; Knap and Doeschl‑Wilson, 2020; Poppe *et al.*, 2020, 2022; Bai and Plastow, 2022). Among the many benefits, they highlight the fact that resilient animals require less attention, thereby decreasing labour and health costs, which represents important economic gains (Knap and Doeschl‑Wilson, 2020). More resilient animals are expected to show minor deviations in performance compared with susceptible ones because they are less impacted by infections or have the ability to rapidly recover from diseases (Mulder and Rashidi, 2017; Berghof *et al.*, 2019b; Knap and Doeschl‑Wilson, 2020). Estimating the ability of an animal to resist or to recover quickly from a disturbance requires a completely novel phenotyping approach based on longitudinal measurements of animal performance before, during, and after a disturbance to provide dynamic trajectories of resilience. As such, multiple traits derived from kinetic production and fitness performance indicators have been explored for an operational measurement of disease resilience (Sandberg *et al.*, 2006; Elgersma *et al.*, 2018; Berghof *et al.*, 2019a, b; Poppe *et al.*, 2020, 2022; Bedere *et al.*, 2022). In these examples, the identification of resilient animals is achieved by measuring the rate of deviation and the speed of return of these key variables to the normal or previous state. Studies might differ in the exact way dynamic resilience indicators are derived but the common underlying assumption is that resilience can be quantified by the scale, pattern, and duration of deviations.

A major advantage is that disease resilience indicators can be quantified in many practical ways based on routine measurements of animal productivity and performance. Ongoing work focuses on improving the heritability of resilience indicators, mainly by better adapting experimental designs to the context of natural disease challenge (Bai and Plastow, 2022). An important question still remains on the effect of breeding for disease resilience on the infection itself, including disease transmission and pathogen evolution. Since resilient animals are able to be productive regardless of the pathogen burden, disease-resilient individuals could increase the spread and persistence of infections (Doeschl-Wilson *et al.*, 2021). Nevertheless, given the contribution of resistance, tolerance, and recovery to disease resilience, one could also expect a reduction of pathogen pressure, hence curbing the arms race between hosts and pathogens.

## Towards an operational measurement of disease resilience in orchards

### Choosing a suitable experimental design for studying disease resilience in fruit trees

A few key elements need to be considered to quantify disease resilience experimentally in orchards. Firstly, orchards must be managed under low phytosanitary protection to allow natural infections to (co-)occur, and because resilience is a dynamic process, monitoring these orchards over several consecutive years is essential. Secondly, experimental orchards must comprise genetically highly diversified accessions, for example by designing a core collection, to capture the phenotypic diversity underlying disease resilience (Frankel and Brown, 1984). In this process, closely related wild species, landraces, and elite cultivars of contrasting geographical origins should be represented and genetically characterized via genotyping or sequencing. Thanks to statistical analyses linking genomic and phenotypic information, such as genome wide association studies (Visscher *et al.*, 2017), it will be possible to decipher the genetic architecture of disease resilience, including the number and position of quantitative trait loci and/or specific genes underlying this trait. The first objective is thus to screen a wide range of genetic diversity to characterize disease resilience mechanisms and their heritability. At a later stage, it may be necessary to investigate the behaviour of resilient candidate varieties in monovarietal orchards.

Finally, given that disease resilience also encompasses the ability of a tree to maintain itself when confronted to a different range of pathogen pressures, studying the response of the same accessions across contrasting environments is also of crucial importance. Of note, for studies focusing on resilience mechanisms *per se*, it can be relevant to provide a fine characterization of the environment in which trees are growing through epidemiological monitoring or envirotyping methods (Xu, 2016). This requires specific instrumentation in the orchards and in this sense, new tools coming from digital agriculture, such as intelligent sensors or remote sensing images, offer attractive perspectives (Lee *et al.*, 2010; Whelan and Taylor, 2013).

To summarize, we propose that setting up a genetically highly diversified, multi-year and multi-site orchard design forms a strong basis for understanding the mechanisms of disease resilience throughout the life of a tree.

### How to monitor disease resilience in orchards: identification of resilience biomarkers

**Fig. 3.**
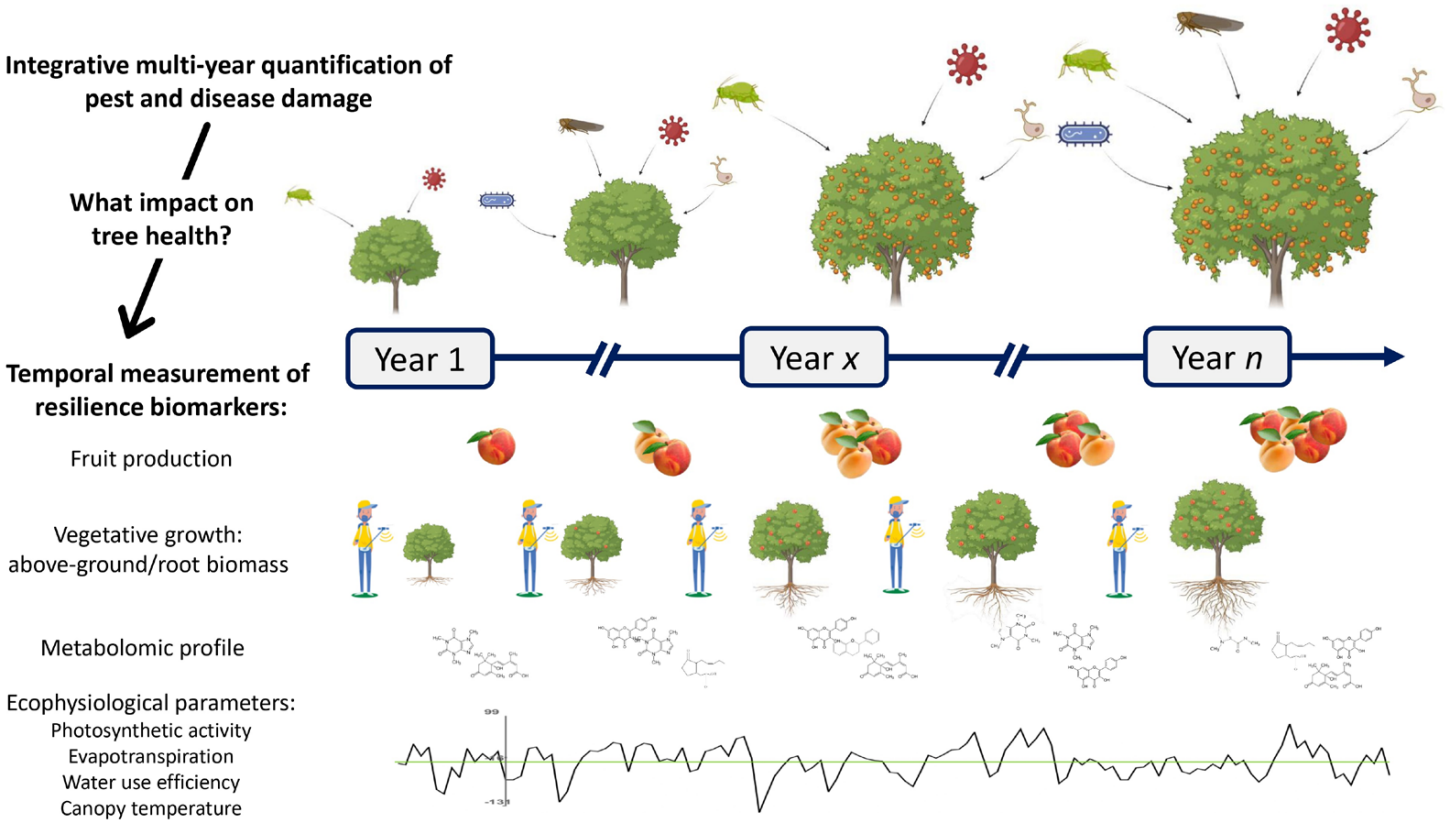
Illustration of resilience biomarkers that could be considered to study the impacts of pests and disease infection on fruit trees under low phytosanitary protection.

Disease resilience is a dynamic trait relying on multiple biological functions that might be difficult to assess accurately in the orchard. Although the classical visual monitoring of disease incidence and severity caused by the diverse source of pathogens (Madden *et al.*, 2017) is an essential step towards characterizing disease resilience, we should search for additional variables reflecting the impacts of infections on tree health over time. Proxy traits of tree performance or health, also called ‘resilience biomarkers’, need to be identified, and importantly, they should not be significantly invasive or destructive. Since disease resilience has not yet been explored in fruit trees, multiple possibilities should be tested and complementary variables might be retained. In the work carried out in livestock, multiple traits derived from kinetic production and fitness performance indicators, such as deviation of body weights of layer chickens over time (Berghof *et al.*, 2019a), fluctuation of milk yield of an individual cow per lactation (Elgersma *et al.*, 2018; Poppe *et al.*, 2020), variation of feed intake and feed duration of grow-finishing pigs (Sandberg *et al.*, 2006; Putz *et al.*, 2019), egg production (Bedere *et al.*, 2022), or daily step count of cows (Poppe *et al.*, 2022), have been identified to illustrate disease resilience. In forestry studies, vegetation indices like normalized difference vegetation index, leaf area index, canopy reflectance, but also defoliation and discoloration are often used in conjunction as tree health indicators (Bannari *et al.*, 1995; Fang and Liang, 2014). Chlorophyll content has also been studied as a possible index of tree health (Talebzadeh and Valeo, 2022). Therefore, many biomarkers of resilience can be considered, such as fruit productivity; vegetative growth through measurements of above-ground or root biomass based on height, leaf or canopy volume, basal area, and branch or trunk diameters (Dobbertin, 2005); ecophysiological parameters such as photosynthetic activity, evapotranspiration, water use efficiency, or canopy temperature (Méthy, 2000; Oerke *et al.*, 2011; Klein *et al.*, 2013); and metabolomic profiles of leaves and sap throughout the entire time of infestation (Castro-Moretti *et al.*, 2021) (Fig. 3). Since the different pests and diseases do not attack the same organs and cause different types of damages, a cumulative measurement of different biomarkers could be relevant to properly illustrate their impact on tree condition. Finally, given that these potential biomarkers are highly linked to environmental conditions, it may be appropriate to correct them by specific cofactors, in particular those linked to climate or to epidemiological knowledge. In apricot, for example, a climatic index of cumulative blossom blight risk has been developed (Tresson *et al.*, 2020) and is already used to prevent misinterpretations of low susceptibility due to the avoidance of contamination related to phenological and meteorological factors.

Once relevant biomarkers are identified and measured over the time, the last step is to derive adequate resilience indicators.

### Proposed indicators to be calculated from dynamic measurements of resilience biomarkers

Multiple approaches have emerged over the last decades to calculate resilience indicators (Ingrisch and Bahn, 2018). Two of them coming from livestock and forestry research seem to be well-suited and offer complementary perspectives to study resilience in fruit trees.

**Fig. 4.**
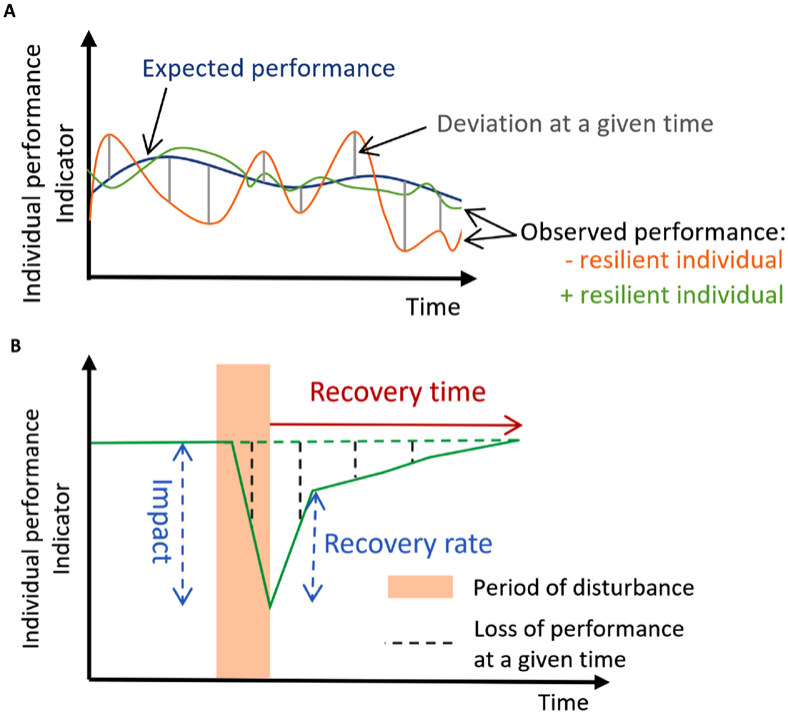
Illustration of resilience indicators proposed by (A) Berghof *et al.* (2019b) to compare disease resilience between livestock animals, and (B) Lloret *et al.* (2011), Thurm *et al.* (2016), and Schwarz *et al.* (2020) to assess the response of forest trees to drought episodes. These indicators are well-suited to study fruit tree disease resilience.

The first one was proposed by Berghof *et al.* (2019b) and sees resilience of livestock in terms of deviations between theoretical and observed performance over a period of time (Fig. 4A). Three resilience indicators can be calculated to illustrate and quantify these deviations: (i) the variance, (ii) the autocorrelation, and (iii) the skewness of deviations over a period of time (Table 1). According to Berghof *et al.* (2019b) the variance of deviations quantifies the impact of disturbances, the autocorrelation gives the length of the impact of disturbances, and skewness is an indication of the direction of disturbances. This method describes particularly well the temporal evolution of resilience biomarkers. We suggest defining a new global resilience indicator based on a combination of variance, autocorrelation, and skewness. The authors further proposed using the slope of reaction norms as an indicator of resilience. A reaction norm describes the pattern of phenotypic expression of a genotype across a range of environmental conditions, which corresponds in our case to a range of different quantities of pathogen disturbance (Cheng *et al.*, 2022). Reaction norm models based on measurement of pathogen load could be helpful for studying the relationship between resilience and its component traits (Knap and Doeschl‑Wilson, 2020). However, calculating reaction norms would require the quantification of pathogen load, which is in practice ill-suited for orchards with multiple natural infections (and re-infections). Given that each disturbance has its own slope, it is also almost impossible to estimate multiple slopes for multiple disturbances occurring at the same time (Berghof *et al.*, 2019b).

**Table 1.** Summary of useful indicators of resilience identified in literature

Indicators	Description	Calculation	Authors
Variance of deviations (σ^2^)	Indication of the impact of disturbances. Captures the severity and duration of environmental perturbations experienced by an individual	$\ln\left(\sigma_{j}^{2}\right)=ln\left(\frac{\overset{n_{j}}{\underset{i=1}{\sum }}{\left(x_{ij}-\;\bar{x_{j}}\right)}^{2}}{n_{j}-1}\right)$ Where *x*_*ij*_ is deviation *i* of the *j*^th ^individual, $\bar{x_{j}}$ is the mean of deviations of the *j*^th^ individual, and *n*_*j*_ is the number of deviation observations of the *j*^th^ individual	Berghof *et al.* (2019a, b)
Skewness of deviations	Indication of the direction of disturbances. Captures the severity of environmental perturbations experienced by an individual	$skewness_{j}=\frac{n_{j}}{\left(n_{j}-1\right)\left(n_{j}-2\right)}\underset{i=1}{\overset{n_{j}}{\sum }}\;{\left(\frac{x_{ij}-\;\bar{x_{j}}}{\sqrt{\sigma_{j}^{2}}}\right)}^{3}$ where *n*_*j*_ is the number of deviation observations of the *j*^th ^individual, *x*_*ij*_ is deviation *i* of the *j*^th ^individual, $\bar{x_{j}}$ is the mean of deviations of the *j*^th ^individual, and $\sigma_{j}^{2}\;$ is the variance of deviations	Berghof *et al.* (2019a, b)
(Lag-one) Autocorrelation of deviations	Indication of the length of the impact of disturbances. Captures the duration (i.e. rate of recovery) of environmental perturbations experienced by an individual	$autocorrelation_{j}=\frac{\overset{n_{j}-1}{\underset{i=1}{\sum }}\left(x_{ij}-\;\bar{x_{j}}\right)\left(x_{\left(i+1\right)\;j}-\;\bar{x_{j}}\right)}{\overset{n_{j}}{\underset{i=1}{\sum }}{\left(x_{ij}-\;\bar{x_{j}}\right)}^{2}}$ where *n*_*j*_ is the number of pairs of subsequent deviations of the *j*^th ^individual, x_ij_ is deviation *i* of the *j*^th ^individual, $\bar{x_{j}}$ is the mean of deviations of the *j*^th ^individual, and $\;x_{\left(i+1\right)\;j}$ is the subsequent deviation of deviation *i* of the *j*^th ^individual	Berghof *et al.* (2019a, b)
Slope of a reaction norm	Indication of resilience in the face of a macro-environmental disturbance. Captures the severity of a macro-environmental perturbation experienced by an individual	The slope of a reaction norm a is estimated based on the trait value of an individual given the level of a disturbance, with a=0 for animals not influenced by the disturbance, a<0 for animals negatively influenced by the disturbance, and the |*a*| value quantifying the impact of the disturbance on the trait	Berghof *et al.* (2019a, b)
Impact	Inverse of performance reduction during the episode (at the time of the peak impact)	$\frac{Performance\;during\;disturbance}{Performance\;before\;disturbance}$	‘Resistance’ in Lloret *et al.* (2011)
Recovery rate	Ability to recover relative to the damage experienced during disturbance	$\frac{Performance\;after\;disturbance}{Performance\;during\;disturbance}$	‘Recovery’ in Lloret *et al.* (2011)
Net change	Capacity to reach pre-disturbance performance levels	$\frac{Performance\;after\;disturbance}{Performance\;before\;disturbance}$ =Impact×Recovery rate	‘Resilience per se’ in Lloret *et al.* (2011)
Relative resilience	Ability to achieve the levels of pre-disturbance performance with respect to the impact during the disturbance	$\frac{Performance\;after-Performance\;during}{Performance\;before\;disturbance}$	Lloret *et al.* (2011)
Recovery time	The time a system needs to return to an equilibrium following disturbance	Time to reach pre-disturbance levels	Thurm *et al.* (2016)
Total performance reduction	Accumulated loss of performance due to the perturbation during the perturbation period plus all the time in the recovery period	$\underset{Begining\;of\;the\;perturbation}{\overset{End\;of\;the\;recovery\;period}{\sum }}\;Loss\;of\;performance\;at\;a\;given\;time$	‘Increment loss due to disturbance’ in Thurm *et al.* (2016)
Average performance reduction	Quantification of the average annual, monthly, etc. perturbation impact	$\frac{Total\;performance\;reduction}{Recovery\;time}$	Schwarz *et al.* (2020)
Average recovery rate	Quantification of how much of the performance reduction could be recovered within one year/month etc.	$\frac{\left(1-Impact\right)}{Recovery\;time\times \left(1-Impact\right)}\times 100$	Schwarz *et al.* (2020)

The second approach was first proposed by Lloret *et al.* (2011) and later completed by Thurm *et al.* (2016) and Schwarz *et al.* (2020). These authors decompose resilience into several indicators to assess the response of forest trees to drought episodes based on tree ring measurements. Illustrated in Fig. 4B and Table 1, these indicators are the following: ‘Resistance’ (subsequently called *Impact*); ‘Recovery’ (subsequently called *Recovery rate*); ‘Resilience per se’ (subsequently called *Net change*); *Relative resilience*; *Recovery time*; ‘Increment loss’ due to disturbance (subsequently called *Total performance reduction*); *Average performance reduction*; and *Average recovery rate*. The first four indicators initially proposed by Lloret *et al.* (2011) were improved by Thurm *et al.* (2016) and Schwarz *et al.* (2020) in order to avoid bias and incorrect interpretations arising from heterogeneity across studies regarding the growth variable types and the lengths of reference periods considered. Based on the measurement of tree-ring width, these indicators have been used in other studies, and have become of great interest in particular for establishing a link between the risk of drought mortality and low resilience to past drought events (DeSoto *et al.*, 2020) or for assessing the influence on trees of successive or prolonged droughts (Schwarz *et al.*, 2020). In their study, and inspired by the work of Nimmo *et al.* (2015), Cantarello *et al.* (2017) used rather similar indicators for assessing forest resilience to different types of disturbances (pulse or pulse+press scenarios). Based on the measurements of different ecosystem services and biodiversity measurements (such as timber volume, aboveground biomass, and total carbon stock), the authors were able to identify thresholds of response according to the type of disturbance. Therefore, these indicators seem to be well-suited to explore disease resilience based on temporal biomarker measurements, and could be applied on pests and diseases damage.

**Fig. 5.**
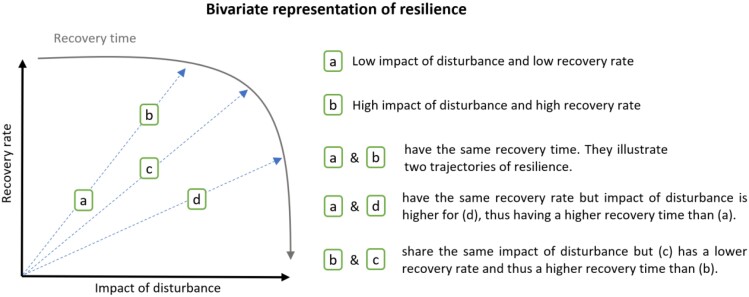
Illustration of the bivariate representation of resilience proposed by Ingrisch and Bahn (2018) by plotting the normalized recovery rate against normalized impact of disturbance in order to define the bivariate space. Recovery time results from the combination of coordinates in the bivariate space. Four examples (a–d) are detailed to describe possible trajectories when searching for resilience. Impact of disturbance is the difference between states at pre-disturbance and maximum impact time; recovery rate is the change of system state per unit time after a disturbance.

To make the most of these indicators, a few authors suggest a bivariate map of resilience based on a joint consideration of resistance and recovery in the system (Hodgson *et al.*, 2015; Ingrisch and Bahn, 2018). This proposal came about in response to the emergence of multiple ways of calculating a metric of resilience leading to diverging conclusions when comparing response trajectories from different ecosystems, different types of disturbances, or different ecosystem state variables. The goal was to obtain a quantitative and comparable assessment of resilience that integrates the major components underlying resilience into a single framework. Thus, Hodgson *et al.* (2015) represent return time in relation to change in state whereas Ingrisch and Bahn (2018) use the normalized impact of disturbance and the normalized recovery rate to define the bivariate space. This vision, illustrated in Fig. 5, could be particularly relevant for a consistent comparison of disease resilience across trees and to account for the different types of pathogen attacks. Besides, this bivariate framework could be useful for assigning the respective roles of resistance, tolerance, and recovery to disease resilience.

## Implementing disease resilience mechanisms into an effective breeding strategy

**Fig. 6.**
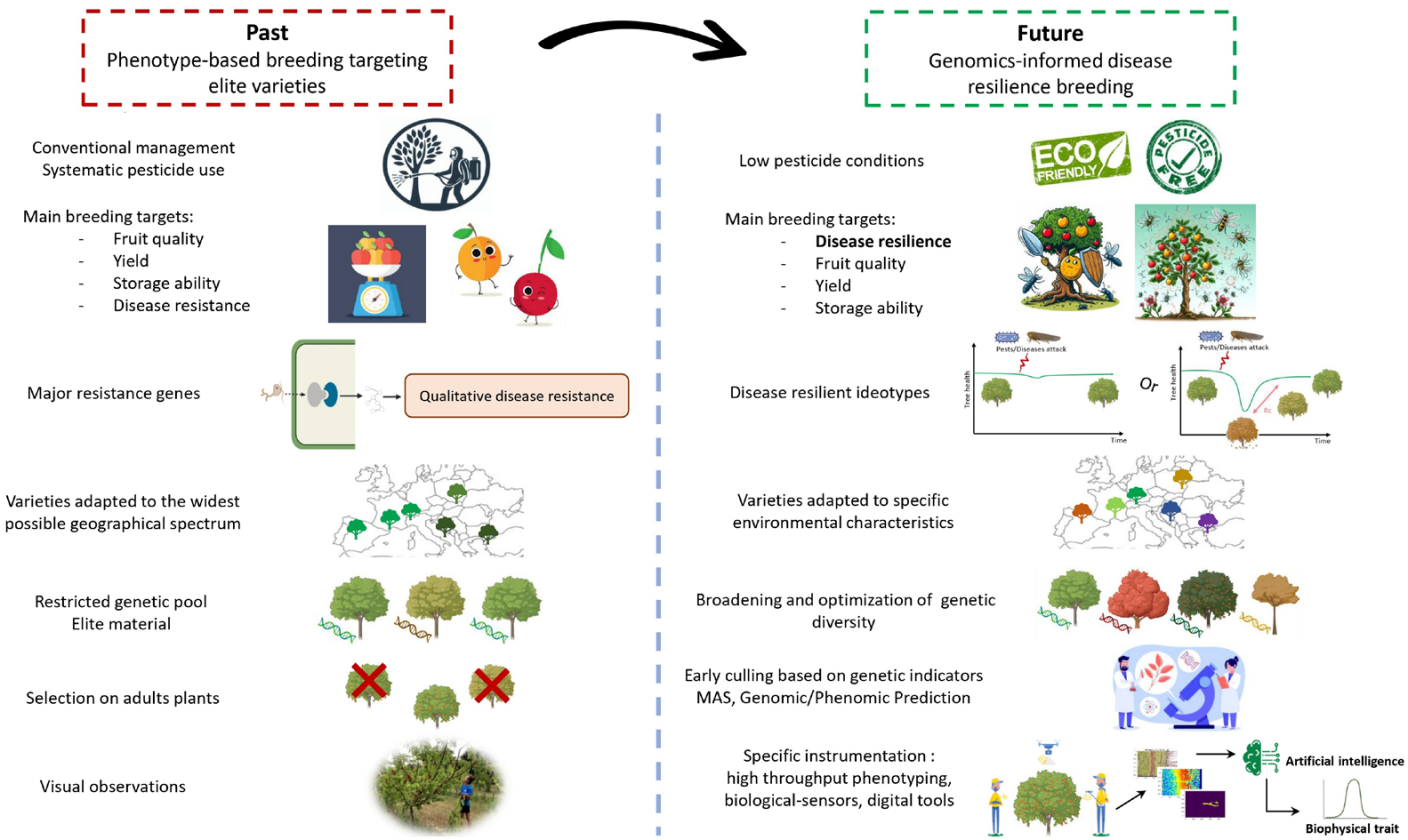
Proposal for future breeding programmes with a description of the major shifts recommended for a transition towards a more sustainable breeding based on resilience breeding.

Breeding for disease resilience means balancing objectives for the simultaneous improvement of tree health in parallel with fruit yield and quality under low phytosanitary input, which calls for the definition of new ideotypes (Debaeke and Quilot-Turion, 2014). Series of resilient ideotypes should be designed to obtain varieties able to cope with the most problematic diseases occurring in the target production area while accounting for further environmental, production, and market requirements (Fig. 6). Considering phenotypic trade-offs, via the inclusion of resilience indicators in the breeding value, is particularly relevant in this context.

For a successful quantification of disease resilience, trees must be observed in challenging and contrasted environments on a multi-year basis. As mentioned previously, given that fruit yield and quality are the highest priorities for breeders, most breeding programmes occur in high-health environments. Therefore, breeding environments need to be completely rethought to allow the spread of pests and diseases and the scoring of resilience indices. An entry step could be the implementation of a ‘multi-disease index’ based on a weighted sum of individual pests and diseases damages. A more advanced step would require temporal and large-scale measurements of relevant biomarkers of resilience from which heritable indicators of resilience could be derived. More research is still required to identify the most suitable biomarkers and to develop easy, non-destructive and inexpensive tools to ensure a regular and precise monitoring of tree health. Specific instrumentation for digital sensing could be implemented to monitor plant stress and growth in a simple way with fixed sensors such as dendrometers (Lamacque *et al.*, 2020). A more sophisticated approach called digital phenotyping, referring to the combination of sensors measuring specific spectral areas (such as visual, infrared, radar), vectors (such as unmanned aerial vehicles [UAVs], robots, satellites) and artificial intelligence (such as deep learning) for the estimation of quantitative phenotypes should also be considered given the recent advances in that field for monitoring diseases (Mahlein *et al.*, 2019). Recent research in urban or forest contexts have shown how to deploy these tools to assess tree health (e.g. Sampson *et al.*, 2003; Degerickx *et al.*, 2018; Vidal and Pitarma, 2019; Sudakova *et al.*, 2021). With the development of new biological sensors and high-throughput phenotyping technologies, temporal data related to tree health might soon make it possible to decipher the genetic and phenotypic architecture of disease resilience. These technologies will likely remain difficult to access for breeders in the short-term. Yet we suggest that flights with standard UAVs equipped with simple RGB cameras could be easily carried out for a regular monitoring of tree health parameters such as chlorophyll or vegetation indices (Zhang *et al.*, 2021). From these measurements, the indicators proposed by Berghof *et al.*, (2019b) can be derived to rank genotypes according to their ability in disease resilience.

It is important to remember that a drastic increase in resilience breeding efficiency should occur once adequate and robust genetic diagnostics are established. With this approach, the breeder can shorten the breeding cycle and/or re-allocate phenotyping efforts towards the screening of more hybrid populations or different traits. To this end, public research is needed to screen large collections representing a vast genetic diversity to identify potential genitors and to develop relevant predictive methods (marker assisted selection, genomic and phenomic selection). These tools are needed to assist breeders in achieving disease resilience goals. Due to the expected genetic complexity of disease resilience traits, genomic selection appears to be highly promising. This method is still underexploited in fruit trees but looks attractive to increase genetic gains for complex traits (McClure *et al.*, 2014). Likewise, phenomic selection—an alternative to genomic prediction based on spectral instead of genetic information—has been developing rapidly in recent years and could be used as a complementary tool (Robert *et al.*, 2022). We emphasize that achieving resilience goals in the near future will not be possible without a close collaboration between public research and private companies enabling the development of adapted phenotypic and genetic tools.

## Conclusion and perspectives: towards a broader consideration of resilience

By being managed in open fields, fruit trees are simultaneously confronted with a large number of biotic stresses over their lifetime, often combined with various types of abiotic stress. This co-occurrence of many potential challenges and stressors has been shown to be particularly destructive (Pandey *et al.*, 2017). As Bai and Plastow (2022) proposed in their review, it could be relevant in the long term to extend our breeding goal from disease resilience to ‘general resilience’ defined as high resilience to different types of perturbations. As general resilience accounts for broad environmental effects, it is particularly adapted for fruit trees affected by fluctuating environments within and between years, but also in the context of global warming where perturbations of different kinds are likely to intensify on broad scales. Therefore, while we describe disease resilience as the first priority for the field today, breeding for generally resilient trees might be the next target in a more distant future.

From the farmer’s perspective, it should not be overlooked that a loss of productivity in a given year may be difficult to tolerate even if production is maximized over the years. To limit the potential inter-annual production variations of resilient varieties and ensure sustainable fruit production, combining resilient varieties with several agro-technical levers will be essential. Among them, we draw attention to cultural practices designed to reduce the incidence of pests and diseases, and to biocontrol (Shaw *et al.*, 2021). In particular, we can mention prophylactic measures to limit entry points and inoculum spread or the use of physical barriers such as rain shelters or insect netting (Gomez *et al.*, 2007; Brun *et al.*, 2023). Biocontrol for its part is based on the use of natural active products, chemical mediators (e.g. sexual confusion or plant defence stimulators) and natural micro- or macro-organism auxiliary (Fauvergue *et al.*, 2020; Shaw *et al.*, 2021; Stenberg *et al.*, 2021). More generally, it is urgent to go beyond highly artificial systems in order to make the most of biological regulation, take advantage of the stimulating and protective effects of the microbiota, and fully express the potential of resilient varieties in order to move towards resilient orchards. This also means mobilizing the different principles of agroecology (Thomas and Kevan, 1993), diversifying cultivated populations, carefully considering the spread of varieties in space and time, using service plants, and if necessary, supplementing these approaches with synthetic products. While it has been demonstrated in some low input trials that agroecology does not systematically have a negative impact on yields (Dittmer *et al.*, 2023), progress is still needed to adapt these practices to each context and to preserve yields within given time and space requirements. We should keep in mind that cultural practices and their interaction with variety choice can impact global orchard resilience, which should be studied in the future in the frame of systemic experiments.

To face multiple biotic and abiotic challenges posed by low input management in fruit tree production, more multidisciplinary work and increased exchanges between stakeholders are needed. Combining a diversity of levers and means of control has great potential to maintain productivity while reducing phytosanitary treatments, making way for environmentally and economically sustainable orchards.
